# Supplementary figures and images for: Aberrant CD3-Positive, CD8-Low, CD7-Negative Lymphocytes May Appear During Viral Infections and Mimic Peripheral T-Cell Lymphoma
Source: Diagnostics (Basel). 2020 Apr 7;10(4):204. doi: 10.3390/diagnostics10040204 (PMC7235783; doi:10.3390/diagnostics10040204)

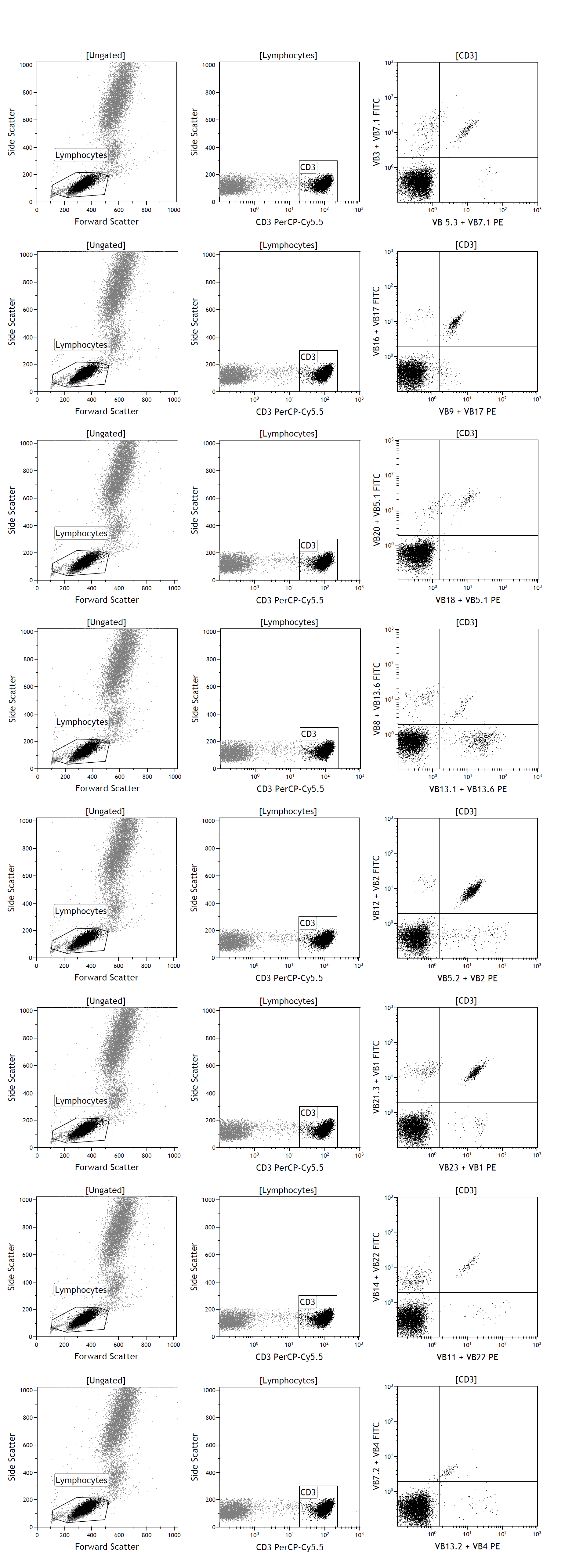

Supplement: Supplementary file 1 [file diagnostics-10-00204-s001.zip › diagnostics-751675-supplementary.bmp]
